# The effect of femoral prosthesis design on patellofemoral contact stresses in total knee arthroplasty: a case–control study with mid-term follow-up minimum 3-year follow-up

**DOI:** 10.1186/s13018-023-04287-2

**Published:** 2023-10-18

**Authors:** Lingce Kong, Wei Lin, Huijun Kang, Ming Li, Kuo Hao, Bo Chang, Fei Wang

**Affiliations:** Department of Orthopedic Surgery, Third Hospital of Hebei Medical University, No. 139 Ziqiang Road, Shijiazhuang, 050051 Hebei China

**Keywords:** Total knee arthroplasty, Patellofemoral joint, Finite element

## Abstract

**Background:**

To investigate the differences in postoperative patellofemoral pressures and patellar tracking during at least three years of follow-up in patients using three prostheses of different designs in total knee arthroplasty (TKA) without patellar resurfacing.

**Methods: Radiographic investigations:**

The study included 401 patients who had a total of 480 knee prostheses implanted without patellar resurfacing. The prostheses used were Genesis II (external rotation design of femoral prosthesis), Triathlon (design with deep trochlear grooves), and Gemini MK II (deepening of trochlear groove and lateral condylar protrusion that closely follows the anatomical shape). The patients' patellar tracking was assessed by measuring patellar tilt and displacement during postoperative follow-up. Furthermore, postoperative knee function and pain were evaluated through range of motion, Knee Society scores (KSS), and Forgotten Joint Score (FJS) to compare the different groups.

**Finite element analysis:**

Constructing a finite element model of the knee joint of a normal volunteer after total knee arthroplasty using different prostheses for nonpatellar replacement. The three models' von Mises stress distribution heat map, peak contact pressure, and patellar transverse displacement were compared at 30°, 60°, and 90°, respectively.

**Results: Radiographic investigations:**

A total of 456 knees of 384 patients were investigated at a 3-year follow-up after TKA without patellar resurfacing. There were no significant differences in patellar tracking between the three groups. Patients with all three prostheses demonstrated favorable clinical outcomes at 3 years postoperatively, with no statistically significant differences in knee scores (91.9 vs 92.3 vs 91.8) or range of motion (127.9° vs 128.5° vs 127.7°) between the groups. However, there was a significant difference between Genesis II and Gemini MK II in the Forgotten Joint Score (59.7 vs 62.4). Patients with persistent postoperative anterior knee pain were present in all three groups (16 vs 12 vs 10), but the incidence was not significantly different.

**Finite element analysis:**

The von Mises stress distribution heat map showed that during flexion, the patellofemoral stresses were mainly concentrated on the lateral side of the prosthesis side, and the contact site gradually shifted downward with increasing flexion angle. At the same time, the peak contact stress of the patellofemoral joint increased with the gradual increase in the flexion angle. Genesis II, with a wider and shallower trochlear groove, showed greater patellofemoral stresses and lateral patellar displacement after TKA without patellar resurfacing. The Gemini MK II with a deeper trochlear groove and slightly protruding lateral condyle is more in line with anatomical design, with smaller patellofemoral joint pressure and better patellar tracking.

**Conclusions:**

In TKA without patellar resurfacing, a prosthesis with a deeper trochlear groove, a slightly higher lateral femoral condyle, and a more anatomically designed knee that better matches the patellar morphology should be a better choice.

## Background

Total knee arthroplasty (TKA) is the ultimate treatment for end-stage osteoarthritis of the knee [1] and is performed by replacing the diseased knee joint surface with an artificial knee prosthesis to achieve pain relief and improved function.

Patellofemoral complications [2] are an important phenomenon after total knee replacement and include anterior knee pain, patellofemoral instability, and loosening and wear of the prosthesis. It has now been shown that patellar maltracking leads to higher patellofemoral contact stresses [3], and higher patellofemoral contact stresses lead to more severe articular surface wear. Among the causes of patellar maltracking, the fit of the patella to the prosthesis [4] is an important factor to consider.

Various factors have been reported to cause patellar maltracking [5], such as the design of the femoral prosthesis, rotation of the femoral prosthesis, and patellar morphology. The main imaging manifestations are lateral patellar tilt and lateral displacement relative to the femur.

However, among the many types of knee prostheses currently available, it is unclear whether femoral prostheses with different trochlear groove designs affect patellar tracking and whether they cause a change in patellofemoral joint contact pressures. Although a study by Leo et al. [6] demonstrated poorer patellofemoral joint function and greater contact pressures in Ortholoc II prostheses with shallower trochlear grooves in clinical postoperative patient follow-up and cadaveric knee replacement, there is still a lack of three-dimensional finite-element analyses to simulate postoperative patellofemoral joint stresses after TKA.

In this study, we will investigate the differences between patellar tracking and patellofemoral joint contact stresses after surgery using different femoral prostheses by analyzing the three-dimensional finite element model of TKA, study the biomechanical effects of femoral prostheses with different trochlear designs on the patellofemoral joint, and analyze the law of stress action to provide a biomechanical basis for further studies on preventing and controlling complications of the patellofemoral joint after TKA surgery. In addition, we will perform radiologic and clinical follow-up investigations to study the differences in postoperative patellofemoral joint function, pain, and alignment in patients with femoral prostheses of different trochlear designs.

We hypothesize that prostheses with deeper trochlea and more anatomically designed knees will result in better patellar tracking and less patellofemoral contact stress postoperatively and that patients with these prostheses will have superior clinical outcomes.

## Methods

### Radiographic and follow-up investigations

**Fig. 1:**
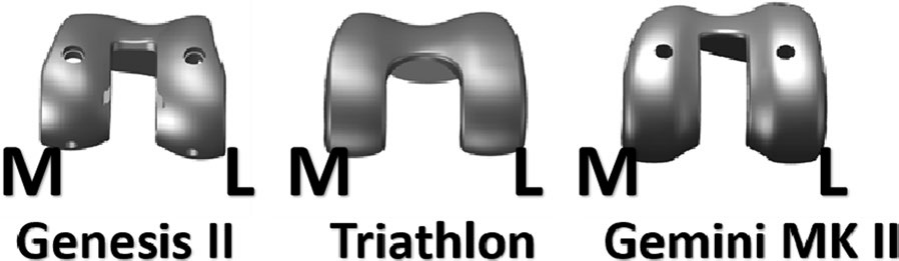
Three knee prostheses with different designs of trochlear grooves. Abbreviations: M, medial; L lateral

A retrospective clinical study was conducted between 2018 and 2020 to determine whether the design of the prosthesis could be a determinant of patellar tracking. The study was approved by the Ethics Committee of the Third Hospital of Hebei Medical University. All patients were fully informed and signed an informed consent form. Patients with osteoarthritis of the knee were selected for the study. The exclusion criteria were patients with severe deformity (valgus or valgus angle more than 15° or flexion contracture more than 20°), rheumatoid arthritis, history of previous high tibial osteotomy, or other previous knee surgery. This study included 480 knees: 160 knees (121 patients) with the Genesis II (Smith &Nephew, America), 160 knees (137 patients) with the Triathlon (Stryker, America), and 160 knees (143 patients) with the Gemini MK II (Link, Germany) (Fig. 1). A total of 480 knees in 401 patients underwent initial TKA without patellar replacement. The three prostheses were implanted alternately without using any randomization procedure.

**Fig. 2:**
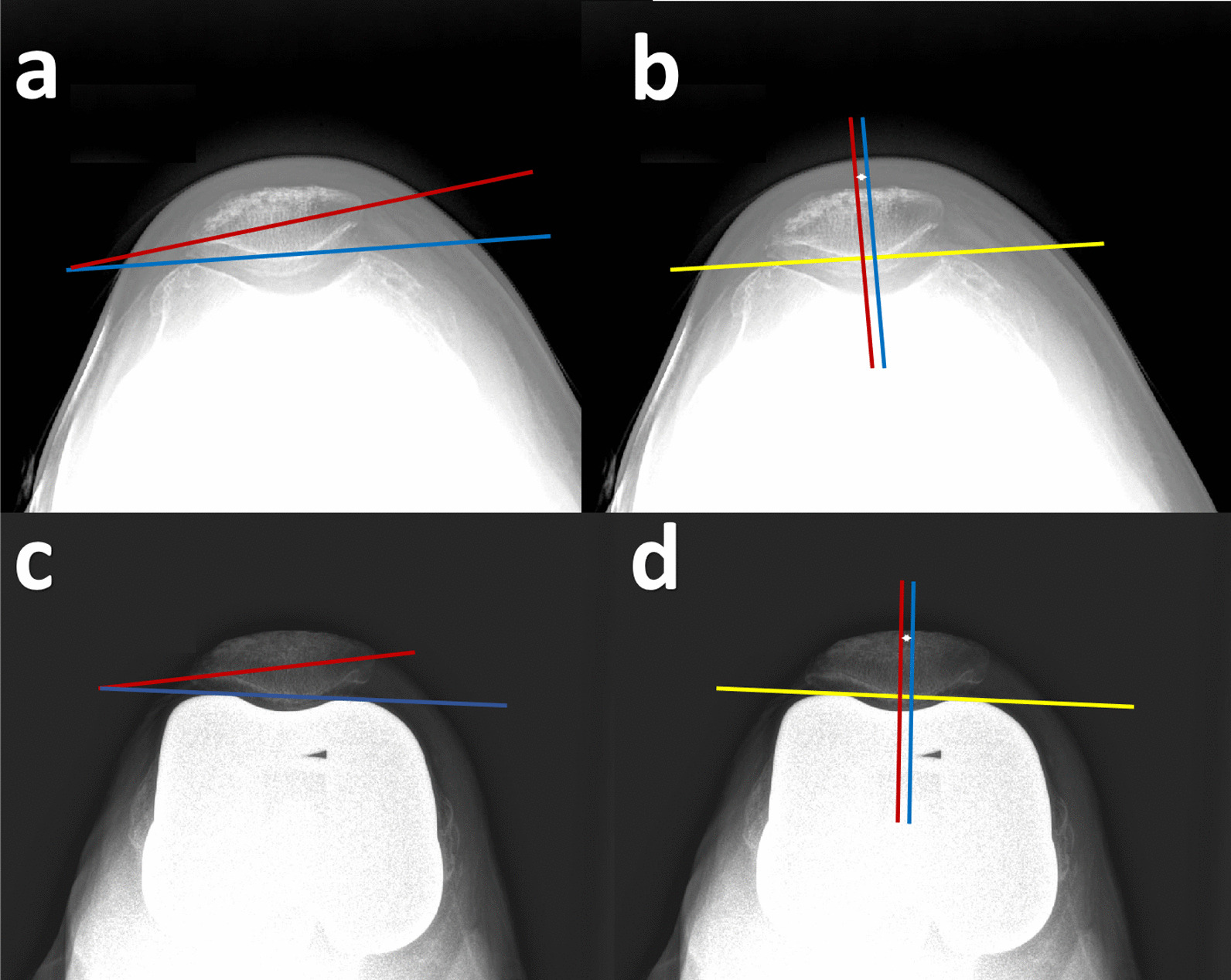
Measurements of preoperative and postoperative patellar tracking. **a**, **c** Preoperative and postoperative patellar tilt: angle between the line through the two anterior femoral condyles (blue line) and the patellar equator line (red line). **b**, **d** Preoperative and postoperative patellar displacement: the line through the anterior condyle of the femur was used as the baseline (yellow line), and the perpendicular lines (red and blue lines) were made from the baseline through the lowest point of the trochlea and the patellar ridge, respectively, and the distance between the two lines was measured

Preoperative patient data, including age, body mass index (BMI), knee range of motion, patellar tilt, and patellar displacement, were recorded. Lower extremity hip-knee-ankle angles were measured using standing anteroposterior radiographs to exclude severe varus or valgus deformities. Axial images of the patellofemoral joint were taken using the Merchant technique with the knee flexed at approximately 45° [7]. Preoperative patellar tilt and displacement were measured using axial radiographs as described by Aglietti et al. [8]. Patellar tilt was defined as the angle between the patellar equatorial line and the through-condylar axis. Patellar displacement was defined as the distance from the trochlear groove to the central patellar ridge, which is the deepest point of the patella relative to the patellar equatorial line (Fig. 2). Lateral patellar tilt or displacement was defined as positive.

All procedures were performed by the same senior surgeon. The three implants used in this study differed in design, mainly in terms of differences in the femoral trochlea. Three different types of prostheses have unique features that help reduce pressure on the patellofemoral joint and improve patellar tracking. The Genesis II prosthesis has an external rotation design and an inclined trochlear groove, while the Triathlon prosthesis has a deep trochlear groove and a relaxing knee extension device. Finally, the Gemini MK II prosthesis has a deepening trochlear groove that closely follows the anatomical shape and a higher lateral condyle for better patellar tracking. The patients were divided into three treatment groups in this study. The surgeon involved in this study had extensive clinical experience. All surgeries are conducted by our senior surgeon and utilize three types of prostheses, resulting in similar procedures. A longitudinal incision of 10–12 cm is made in the middle of the knee, and a medial approach to the patella is used to cut the joint capsule for opening and closing. The patella is then everted, and the anterior cruciate ligament and meniscus are removed. Osteophytes and proliferative synovium are also removed from the distal femur and proximal tibia to fully expose the tibial plateau. A tibial bone marrow external positioning rod is then installed, and the tibial plateau is tilted back 3–5° for osteotomy. The posterior cruciate ligament is preserved throughout the procedure. Distal femoral osteotomy is performed with external rotation of 3° and valgus of 5–7°, measured at the knee extension position to balance the gap. The external rotation osteotomy is done with reference to the posterior condylar line and the intercondylar line. After cleaning the posterior osteophyte, the joint capsule is released, and the prosthesis is tested for stability. Once stability is confirmed, the prosthesis is fixed with bone cement and a polyethylene gasket is installed. Then release the tourniquet, thoroughly stop the bleeding, rinse the joint cavity, and sew the layers together.

**Fig. 3:**
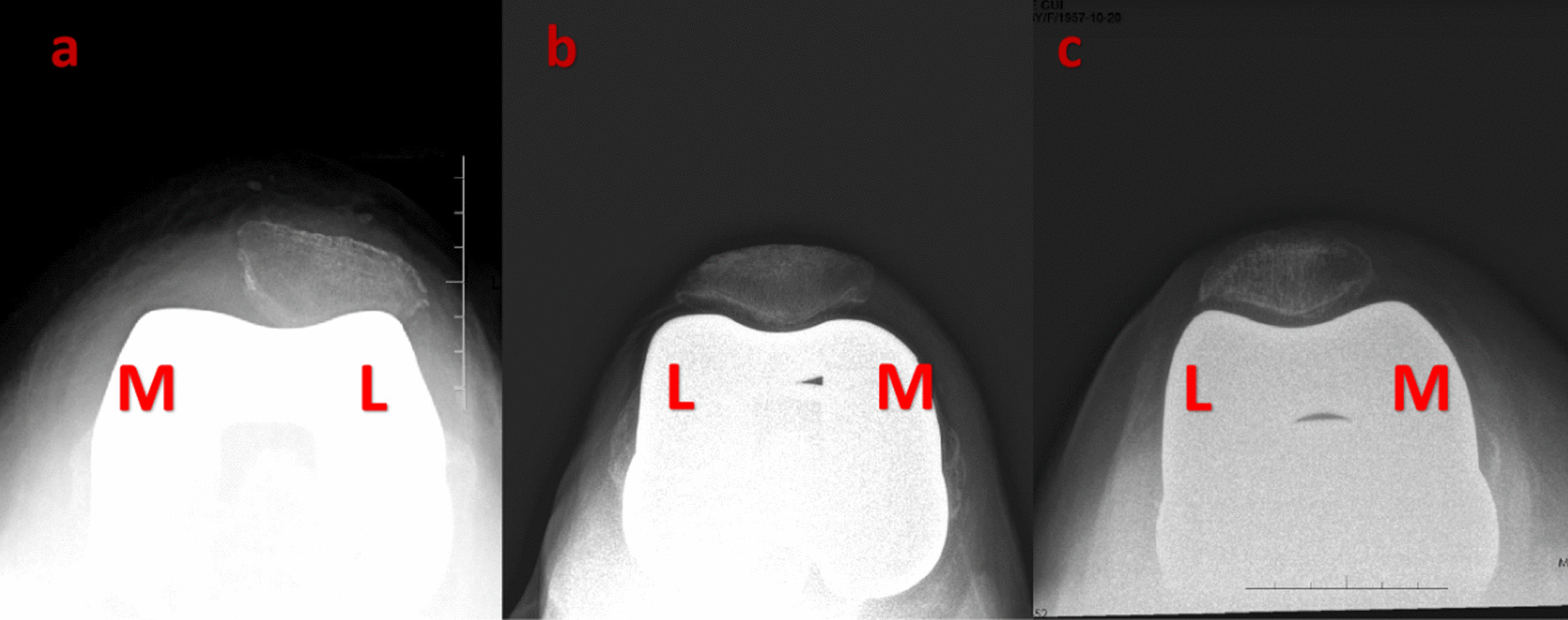
Postoperative follow-up axial patellar X-rays. **a**. Genesis II **b**. Triathlon **c**. Gemini MK II. Abbreviations: M, medial; L lateral

Patients were followed up at 1 month, 3 months, 6 months, and 1 year postoperatively and annually thereafter. Postoperative patellar tilt and displacement were measured according to the method described by Gomes et al. [9] (Fig. 2). Postoperative patellar tilt and displacement were compared among the three groups of patients (Fig. 3). The degree of improvement in postoperative patellar tracking is represented by the difference between preoperative and postoperative measurements. The range of motion, KSS, and FJS were also recorded and compared, as well as for patients with persistent postoperative anterior knee pain.

#### Data measurement

The data about the preoperative and postoperative patellar tilt and displacement were measured by three senior independent surgeons who strictly followed the blinding principle to calculate the intraclass correlation coefficient (ICC). A value of ICC ≥ 0.8 was considered good and ≥ 0.9 excellent.

## Statistical analysis

Statistical comparisons of clinical outcomes were performed using SPSS version 26.0 software (SpSS Inc., Chicago, IL). Means and standard deviations were used to describe continuous variables, and all continuous variables conformed to a normal distribution with the chi-square test. The statistical difference in preoperative and postoperative measurements was calculated using paired t tests. Differences between continuous variables in each group were calculated using one-way ANOVA, and the incidence of anterior knee pain among the three groups was verified using chi-square tests. Statistical significance was defined as *P* < 0.05.

## Finite element study

### Study subject and acquisition of imaging data

A healthy adult male was selected. Other knee diseases, such as knee deformity, knee trauma, and tumor, were excluded by history, physical examination, bilateral CT, and MRI of the knee. Deformities of the hip and ankle joints were also excluded by full-length X-ray examination of the volunteers' lower extremities. Informed consent was obtained and signed by the volunteers. The knee of the volunteer was scanned using a 16-row double helix CT (Siemens, Germany) and a 3.0 T MRI (Siemens, Germany). The knee joint was naturally straightened, and the scanning area was 10 cm above and below the knee joint gap. CT images were mainly used to observe the bone tissues, and MRI images were mainly used to observe the soft tissues, such as cartilage, meniscus, and ligaments, which indicated that there was no relevant tissue damage in the knee of the volunteer.

### Constructing the TKA finite element model

The original data were collected in DICOM format and imported into Mimics Medical 21.0 software (Materialise, Belgium). The "Mask" file was generated using the threshold segmentation function and area growth function of the software to form the 3D surface models of the distal femur, proximal tibia, proximal fibula, patella, patellar ligament, quadriceps tendon, and medial and lateral collateral ligaments; it was converted into an STL file and imported into Geomagic 2021 (Geomagic, USA), and smooth each model to generate cartilage models of the femur, tibia, and patella; STEP format files of the bony structures, ligaments, tendons, and cartilage structures were imported into Solidworks 2021 (Dassault, France) and assembled into each component to build the original knee joint model.

**Fig. 4:**
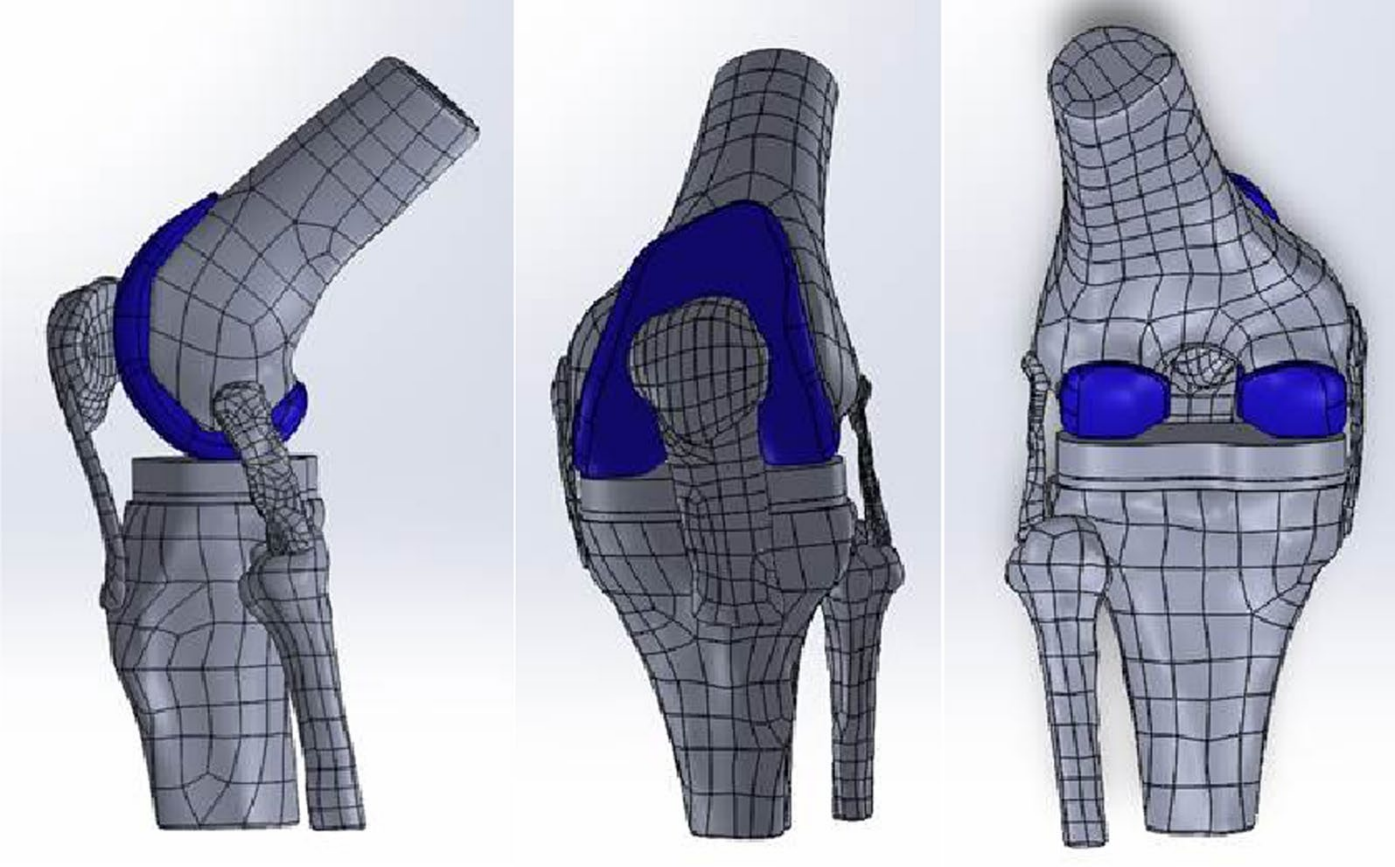
Finite element of TKA

Based on the finite element model of the normal knee joint, the appropriate size of the artificial prosthesis was selected, and the CT data of the prosthesis were extracted by Mimics Medical 21.0 software to obtain the STL files of the femoral component (cobalt–chromium–molybdenum alloy), the tibial component (titanium alloy) and the polymer polyethylene spacer of the TKA prosthesis and then imported into Geomagic Studio 12.0 software for surface treatment and exported to the STEP format file. The prosthesis was assembled with the knee using Solidworks 2021 (Dassault, France). First, the unwanted soft tissues were eliminated; second, the femoral and tibial prostheses were loaded in a “cut-out” position to simulate the clinical surgery. Finally, the complete knee model was saved as an x-t file and imported into ANSYS 2021 R1 (ANSYS, USA) for the next series of operations (Fig. 4).

### Mesh division and material property assignment settings

After the x-t file is imported into ANSYS software, the material properties of the required analysis model are edited and recorded in advance in the Engineering Data of the static module. The specific material properties used for the finite element are shown in Table 1. Second, the model is meshed, and the ten-node tetrahedron type is selected. The specific control dimensions of the model mesh are shown in Table 2. The current model mesh is based on previous literature as references [10–12], combined with actual data conditions.

**Table 1:** Finite element material property assignments

Materials	Modulus of elasticity /MPa	Poisson's ratio
Bone	16,600	0.3
Articular cartilage	5	0.46
Ligament	467	0.46
Patellar tendon	778	0.46
Quadriceps muscle group	413	0.29
Cobalt–chromium–molybdenum	125,000	0.36
Spacer	1950	0.43
Tibial metal brace	227,000	0.31

**Table 2:** Finite element mesh division

Model	Mesh size/mm	Nodes	Elements
Bone	3	69,708	39,590
Articular cartilage	0.5	49,279	25,270
Ligament	2	41,383	22,490
Femoral component	3	17,433	9540
Spacer and Tibia component	4	7776	3125

### Model force analysis

According to previous studies in the literature [12–14], the lower-end surfaces of the tibia and fibula at the distal end of the knee joint were constrained to limit the load, a 400 N tension force was applied to the upper edge of the quadriceps muscle to simulate muscle lifting and pulling, and a 300 N vertical load was added to the upper-end surface of the proximal end of the femur to simulate half of the normal human body weight. The TKA finite element model was designed at flexions of 30°, 60°, and 90°. In order to promote optimal function of the patellofemoral joint, our approach involves first fine-tuning the flexion angle of the knee joint, and then making any necessary adjustments to the ligaments' starting and ending positions based on anatomical structure. We ensure that boundary conditions remain consistent across all angles. Our force analysis involves subjecting each model to a static stress situation lasting one second.

### Observations

1. von Mises stress distribution heat map. The stress level is indicated by different colors and displayed in each finite element model, which intuitively reflects the size and distribution of the stress on the patellofemoral joint surface.
2. Peak contact pressure of the patellofemoral joint. Under the conditions of different knee prostheses and different flexion angles, the peak contact pressure of the patellofemoral joint was recorded.
3. Patellar transverse displacement. The change in patellar displacement relative to the initial position under different flexion angles was recorded for models using different prostheses, and transverse component displacement was used as an index for evaluating patellar tracking.

## Results

### Reliability of measurement

The intraobserver ICC values for preoperative patellar tilt, preoperative patellar displacement, postoperative patellar tilt, and postoperative patellar displacement were 0.81, 0.82, 0.92, and 0.86, respectively, indicating strong intraobserver reliability for these measurements.

### Radiographic and follow-up investigations

A total of 456 knees (Genesis II prosthesis; 150 knees, Triathlon prosthesis; 150 knees, Gemini MK II prosthesis; 156 knees) underwent a 3-year postoperative follow-up survey. The preoperative demographics of the patients are shown in Table 3. There were no significant differences in preoperative demographics among the three groups of patients.

**Table 3:** Patient demographics

	Genesis II	Triathlon	Gemini MK II	P
Age (years)	65.3 ± 7.5	66.3 ± 8.3	66.7 ± 7.6	0.259 (n.s)
BMI (kg/m^2^)	25.6 ± 2.9	26.1 ± 3.0	25.7 ± 2.9	0.270 (n.s)
Patellar tilt (°)	9.2 ± 3.9	9.9 ± 3.7	9.3 ± 3.4	0.185 (n.s)
Patella displacement (mm)	3.2 ± 1.8	2.8 ± 1.8	3.1 ± 1.7	0.197 (n.s)
Knee range of motion (°)	108.1 ± 12.9	105.7 ± 12.8	107.8 ± 13.6	0.228 (n.s)

At the 3-year postoperative follow-up, all three groups of patients showed significant improvement in patellar tilt and displacement compared to preoperative measurements (Table 4). However, there was no significant statistical difference between the three groups in terms of postoperative patellar tilt, patellar displacement, and degree of improvement. (Table 5). There was no significant difference between the three groups in the postoperative KSS or the range of motion. However, in the FJS, the Gemini MK II had better results than the Genesis II (Table 5). At the final follow-up, there were no serious complications related to the patellofemoral joint, such as patellar dislocation, fracture, or loosening, but the number of patients with persistent anterior knee pain in each of the three groups was as follows: Genesis II prosthesis; 16 knees, Triathlon prosthesis; 10 knees, Gemini MK II prosthesis; and 12 knees.

**Table 4:** Preoperative versus postoperative patellar tracking

patellar tracking	Genesis II		p	Triathlon		p	Gemini MK II		p
	Preoperative	Postoperative		Preoperative	Postoperative		Preoperative	Postoperative	
Tilt (°)	9.2 ± 3.9	8.1 ± 4.5	0.001	9.9 ± 3.7	7.1 ± 5.2	0.036	9.3 ± 3.4	7.7 ± 4.8	< 0.001
Displacement (mm)	3.2 ± 1.8	2.3 ± 2.2	< 0.001	2.8 ± 1.8	2.2 ± 1.5	0.001	3.1 ± 1.7	2.1 ± 1.7	0.003

**Table 5:** Patient postoperative follow-up and imaging measurements

	Genesis II	Triathlon	Gemini MK II	P
Patellar tilt(°)	8.1 ± 4.5	7.1 ± 5.2	7.7 ± 4.8	0.195 (n.s)
Patella displacement (mm)	2.3 ± 2.2	2.2 ± 1.5	2.1 ± 1.7	0.627 (n.s)
Tilt improvement (°)	1.1 ± 6.2	2.8 ± 6.7	1.5 ± 5.7	0.055 (n.s)
Displacement improvement (mm)	0.7 ± 2.6	0.6 ± 2.2	1.1 ± 2.5	0.216 (n.s)
Knee scores	91.8 ± 2.9	92.3 ± 2.9	91.9 ± 2.9	0.345 (n.s)
Function score	84.8 ± 5.1	85.3 ± 5.4	85.1 ± 5.2	0.777 (n.s)
Knee range of motion (°)	127.7 ± 10.7	128.5 ± 10.5	127.9 ± 10.6	0.819 (n.s)
Forgotten Joint Score	59.7 ± 10.5*	61.6 ± 9.8	62.4 ± 8.9*	**0.043**
Anterior knee pain	16	10	12	0.428 (n.s)

Bold represents a *p*-value < 0.05, with statistical differences

*Represents statistical significance between the two groups

### Finite element analysis

#### von Mises stress distribution heat map

**Fig. 5:**
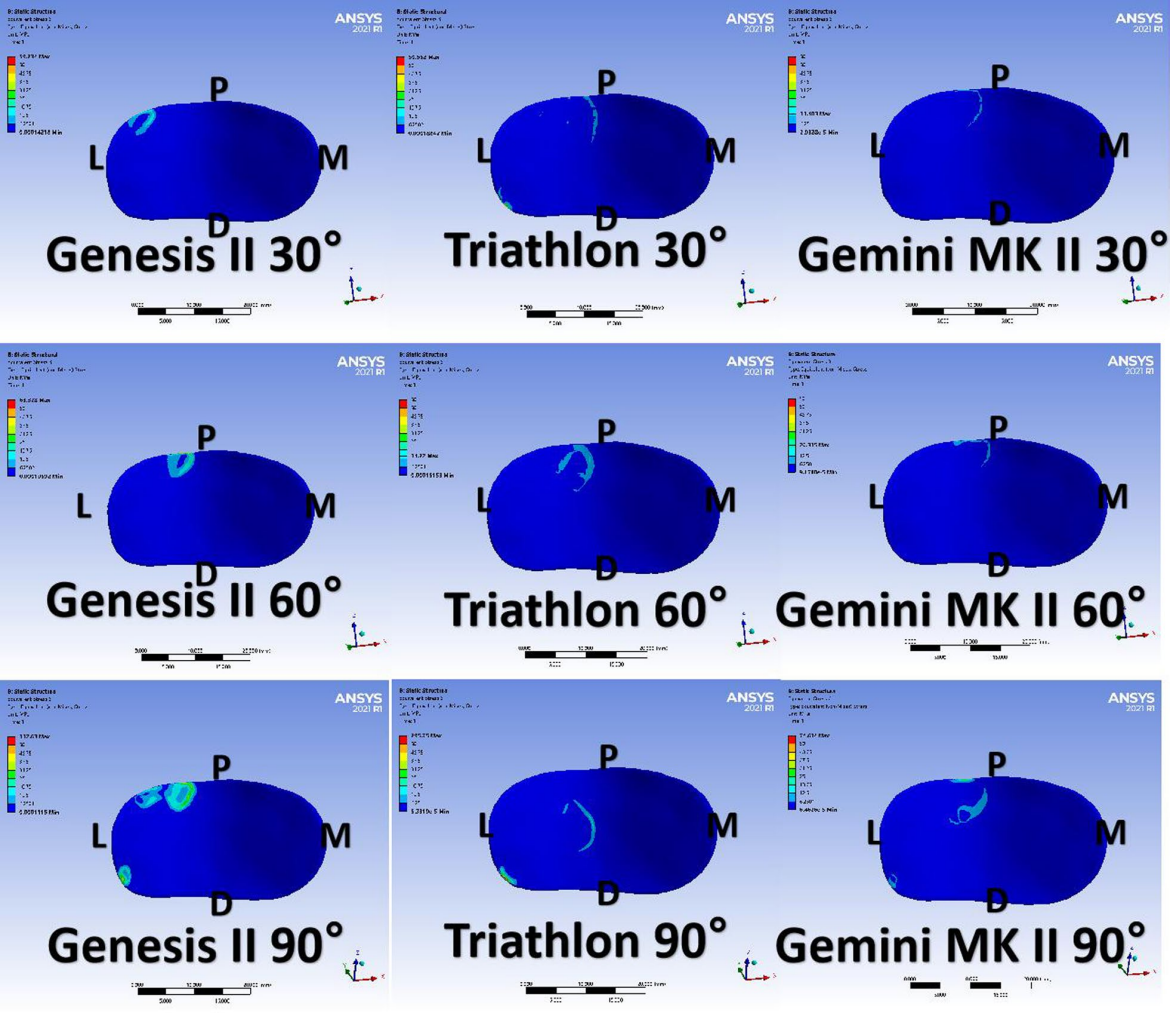
von Mises stress distribution heat map of different prosthesis models at different flexion angles after TKA. Abbreviations: M, medial; L lateral; P, proximal; D, distal

The von Mises stress distribution heat map of the TKA model (Fig. 5) showed that during knee flexion, the patellofemoral joint stresses were mainly concentrated on the lateral side of the prosthesis side, and the contact site gradually shifted downward with increasing flexion angle. Meanwhile, the patellofemoral contact stress increased with the gradual increase in flexion angle.

#### The peak contact pressure of the patellofemoral joint

The effects of flexion angle on the peak contact pressure of the patellofemoral joint varied consistently in the TKA finite element models established based on data from three different types of prostheses (Tables 6 and 7). In the same joint prosthesis model, the patellofemoral joint peak contact pressure tended to increase as the knee flexion angle increased. At the same flexion angle, the patellofemoral joint stress was greater in Genesis II than in the other two prostheses.

**Table 6:** Peak patellofemoral contact pressure (e)

	30° (Mpa)	60° (Mpa)	90° (Mpa)
Genesis II	110.23	118.22	235.71
Triathlon	86.689	90.393	175.14
Gemini MK II	50.213	76.829	126.22

**Table 7:** Peak Patellofemoral Contact Pressure (Patellar Side)

	30° (Mpa)	60° (Mpa)	90° (Mpa)
Genesis II	86.51	100.51	116.24
Triathlon	43.802	56.646	76.244
Gemini MK II	19.936	38.543	51.038

#### Patellar transverse displacement

In the Triathlon and Gemini MK II articulating prosthesis models, lateral patellar displacement increased with increasing flexion angle (Table 8). However, in the Genesis II prosthesis model, patellar displacement was slightly less at 60° of flexion than at 30°. Additionally, in each of the flexion angle models, the patellar displacement was greater in Genesis II than in the other two prostheses.

**Table 8:** Lateral displacement of the patella

	30° (mm)	60° (mm)	90° (mm)
Genesis II	6.8358	3.3382	12.15
Triathlon	2.9015	3.081	6.4871
Gemini MK II	1.2301	2.231	4.7213

## Discussion

In the clinical follow-up part of this study, we compared the clinical outcomes of TKA using three different prostheses. The design of the prosthesis was found to have no significant effect on the interim follow-up, which lasted three years, either in terms of imaging or in terms of the patient's knee function and pain. The Gemini MK II was superior to the Genesis II only in terms of the FJS. However, the finite element study demonstrated that Genesis II, with its wider and shallower sulcus, had greater patellofemoral contact pressures and relatively poor patellofemoral tracking after surgery.

TKA is the ultimate treatment for advanced knee disease, and its main objectives are to reduce joint pain, restore the alignment of the lower limb lines of force, and ensure the stability of the knee [15]. Anterior knee pain is one of the major causes of unsatisfactory postoperative results in patients with TKA, and in severe cases, even revision surgery is required to treat it. TKA without patella replacement needs to take into account the fit of the patella to the articular prosthesis; poor patellar tracking, uneven stress distribution, and excessive stress in the patellofemoral joint caused by poorly fitted knee prostheses [6] can lead to the occurrence of postoperative anterior knee pain symptoms.

The design of the femoral prosthesis is an important factor influencing the clinical outcome. The results of the finite element part of this study confirmed our hypothesis that femoral prostheses with a deeper trochlear groove and a higher lateral condyle have better patellar tracking and patellofemoral alignment and possess less contact stresses in TKA with an unreplaced patella. Unreplaced native patella may show earlier patellar and trochlear wear and symptoms of anterior knee pain due to imbalanced pressure distribution and localized pressure buildup when applied to prostheses with wider and shallower trochlear grooves, which may explain the slightly lower postoperative FJS in patients with the Genesis II prosthesis. On radiology, patients with femoral prostheses with wider shallow trochlear grooves showed greater lateral patellar tilt and displacement, suggesting slightly worse patellar tracking postoperatively, although they did not differ significantly in radiological measures. Differences in patellofemoral joint pressures and patellar tracking may not have caused excessive prosthesis wear in patients with mid-term follow-up. However, this effect may become progressively more apparent as the prosthesis service life increases. This was one of the aims of our finite element study.

The Forget Joint Score is used to evaluate joint awareness in patients undergoing TKA [16]. In our sample, almost no patients completely forgot their knees after TKA. This suggests that patients often take longer to adjust to a knee that has been replaced. Improved joint forgetfulness is an important indicator of satisfaction after TKA, and scholars have recommended routine follow-up of joint awareness after TKA [17, 18]. The current approach to assessing prognosis often overlooks the subjective experiences of some patients. Our study found that using the Gemini MK II, which has a smaller sulcus angle and a more prominent lateral condyle, resulted in better FJS during postoperative follow-up compared to the Genesis II. This indicates that correcting patellar tracking can reduce knee awareness in patients, helping them better adjust to their replaced knee.

The need for patellar replacement in total knee arthroplasty has not been accurately determined in previous studies [19–21]. The design of femoral prostheses is mostly matched with well-aligned patellar prostheses, and the efficacy of patellar surface replacement in early TKA was significantly higher than that of the nonreplacement group, leading to the widespread practice of simultaneous intraoperative patellar replacement. Moreover, a higher incidence of postoperative anterior knee pain in TKA without patellar replacement has been reported in some studies [20, 22], but factors such as patellar morphology and implant type were not adequately considered in these studies. Modern designs of prostheses have the advantage of being patellofemoral friendly, and patellar replacement or not does not make a significant difference in the clinical outcome of patients [23, 24]. In some studies [19, 25], patients without patella replacement showed better clinical outcomes in modern well-designed prostheses. As prostheses are modernized, choosing a more patellofemoral-friendly prosthesis may improve the clinical outcome of TKA.

Previous studies have shown that the fit of the femoral prosthesis and patella affects the clinical outcome of the patellofemoral joint [26]. When the patellar and femoral prosthesis fit is poor, it may lead to severe wear and tear, especially in patients who have not undergone patellar replacement and may lead to more severe symptoms of anterior knee pain. The fit of the prosthesis to the patella is an important factor affecting postoperative functional recovery. Previous studies [27, 28] using low contact stress prostheses found that the presence or absence of patellar replacement did not have a significant effect on the incidence of postoperative anterior knee pain and difficulty in ascending and descending stairs because the morphology of the anterior edge of the femoral prosthesis was closer to that of the normal human femoral anatomy. The patellar tracking is similar to that of the natural condition, and there is no significant difference in the fit of the patellar prosthesis and native patella to the femoral prosthesis. In recent years, patellofemoral-friendly prostheses have demonstrated a good fit to the patient's own patella, with deepened trochlear grooves and enlarged lateral patellar facet support to better match the native patella [6]. Intraoperatively, polishing the patella is possible, thereby making the patellofemoral joint more compatible. None of the patients we included had patellar surface replacement, and the finite element analysis we used did not replace the patellar surface to ensure that our study focused on the fit of the native patella to the femoral prosthesis.

Rotation of the femoral prosthesis also affects patellar tracking and patellofemoral joint pressures. One study found that internal rotation of the femoral prosthesis in TKA was associated with anterior knee pain, knee instability, and stiffness [29]. Its optimal external rotation angle is 3°, and an overly internally rotated femoral prosthesis increases patellofemoral joint contact stress [30], which is an important risk factor for anterior knee pain and prosthesis wear after TKA. It has also been shown [31] that excessive external rotation of the femoral prosthesis similarly leads to elevated patellofemoral joint contact stresses, so improving patellar tracking by altering femoral prosthesis rotation is not entirely sufficient. It is necessary to combine the adjustment of femoral prosthesis rotation with design improvements to obtain better patellar tracking.

Patellofemoral joint symptoms after TKA vary between Wiberg classifications. Previous studies [1, 32, 33] have shown that Wiberg type III patella with small patellofemoral surface angles results in smaller patellofemoral joint contact areas and greater contact pressures. Additionally, the uneven patellofemoral joint pressure distribution due to the different contact areas of the medial and lateral patellofemoral surfaces is the main cause of patellofemoral joint pain. Since Wiberg type II patellae are the most common in the population [1], Wiberg type II patellae were used for finite element simulations in this study to accommodate the patellar morphology of the majority of the population and to better model its effect on patellofemoral joint pressure.

In this study, three-dimensional finite element reconstruction was utilized to simulate the change in patellofemoral contact pressure after TKA, and it was seen that the patellofemoral contact pressure was higher in the prosthesis with a shallower and wider trochlear groove than in the other two groups. The deeper trochlea and extended lateral articular surface allowed the prosthesis to reduce the patellofemoral contact pressure to more closely resemble that of a normal knee. Usually, if the angle of the trochlear groove of the prosthesis is higher than that of the normal femoral trochlear groove [1], the patella may tilt outward and move laterally. We believe that prostheses with smaller sulcus angles and deeper trochlea have better patellar confinement, resulting in better patellar tracking and more even patellofemoral pressure distribution.

In addition, the patellofemoral contact pressure varies at different flexion angles. As the flexion angle increased, the pressure also increased, which is consistent with previous findings [33]. The disparity between the patellofemoral contact areas of the different femoral prostheses was also more pronounced at larger flexion angles, and this phase is of greater significance for patellofemoral joint wear. Because of the increased pressure base during flexion, even small changes in the contact area can cause increased wear at the contact site [34]. Therefore, the incidence of patellofemoral joint syndrome after TKA is significantly higher in prostheses that are relatively mismatched to the patella, and the symptom of pain in the patient going up and down stairs is more prevalent.

Our study demonstrated that mid-term follow-up showed favorable clinical outcomes in three different types of knee prostheses with different trochlear designs. However, the design of the prosthesis should be considered a key factor influencing patellar tracking. Individuals with femoral prostheses featuring wider and shallower trochlear grooves experienced an increase in postoperative patellar tilt and displacement, despite no significant difference in these parameters among the three groups. During our research on finite element analysis, we discovered notable variations in patellofemoral pressure among models that employed different prostheses. Although the current prosthetic designs have exhibited favorable outcomes in patients' mid-term follow-ups, our analysis suggests the possibility of potential patellofemoral damage. It is possible that this pressure difference may not have any impact on the occurrence of postoperative patellofemoral joint syndrome in the early and mid-stages, but it may promote prosthesis wear in the long run. However, we believe that further validation in a larger study is needed to better investigate the changes in patellar tracking with different prostheses.

The significance of this study is that, by means of three-dimensional finite element reconstruction, it was confirmed that femoral prostheses with smaller sulcus angles and deeper trochlear grooves resulted in better patellar tracking and better patellofemoral pressure distributions after TKA, which can provide a theoretical basis for the design of future knee prostheses. This study has some limitations. 1. Patellofemoral kinematics has a complex mechanism. As measured parameters of patellar tracking before and after surgery, patellar tilt and displacement do not fully represent patellofemoral kinematics. 2. Although malrotation of the femoral component by overrotation has a significant effect on patellofemoral joint contact pressure and patellar tracking, this factor was not taken into account in our clinical study or finite element analysis. 3. Although a finite element study was conducted, the mid-term follow-up did not fully reflect the results. To draw conclusive findings, a longer follow-up may be necessary.

## Conclusion

In TKA without patellar resurfacing, a prosthesis with a deeper trochlear groove, a slightly higher lateral femoral condyle, and a more anatomically designed knee that better matches the patellar morphology should be a better choice.

## Data Availability

The datasets used or analyzed during the current study are available from the corresponding author through emails on reasonable request.

## Abbreviations

TKA: Total knee arthroplasty
KSS: Knee Society scores
FJS: Forgotten joint score
